# Epidural Blood Patch Performed Immediately After Dural Puncture and Epidural Drug Administration

**DOI:** 10.7759/cureus.16634

**Published:** 2021-07-26

**Authors:** Polymnia Galanou, Theofilos Tsoleridis, Savvas Tsoleridis

**Affiliations:** 1 Anesthesia and Pain Treatment Unit, General Hospital of Rhodes, Greece, Rhodes, GRC

**Keywords:** case report, autologous blood patch, epidural, post dural puncture headache, lumbalgia

## Abstract

The epidural blood patch (EBP) is a solution for persistent headaches following an accidental dural puncture. We report three cases where EBP was performed immediately after dural puncture combined with drug administration for lumbago. To the best of our knowledge, no similar cases have been reported in the literature.

The patients provided their informed consent, and all standard protocols were followed. At the moment of the dural puncture, all the patients manifested severe headaches, nausea, vomiting, and profuse sweating. A second epidural puncture was performed at a higher intervertebral space, followed by drug administration and 20 mL of autologous blood. All the patients improved gradually, while their headaches vanished after 30-35 minutes. The patients were kept in for monitoring and released the following day with specific instructions and daily phone contact for a week without any complications. Their lumbago symptoms regressed.

The possibility of pneumocephalus was excluded because of the patients’ symptomatology. The risk of high or total spinal anesthesia due to local anesthetic leakage subarachnoidally was countered with close monitoring of the patients. EBP complications include failure, postdural-puncture headache worsening by an accidental additional dural tear(s), back pain, and infection. All patients were instructed to report any symptoms immediately. EBP executed immediately after dural puncture seems to relieve headache fast and does not appear to impede epidural analgesia.

## Introduction

Epidural drug administration is a common treatment option for patients with lumbalgia sciatica (lumbago) and offers rapid analgesia. Patients usually prefer it as an alternative to spinal surgery if they cannot undergo an operation for technical or motivational reasons. The efficacy of epidural analgesia has been well documented, and it has been proven to reduce chronic pain and physical and emotional impairment [1,2].

However, possible complications may occur. Accidental puncture of the dura mater while performing epidural analgesia is a common complication (59-85%). The perforation from the needle results in cerebrospinal fluid (CSF) leaking into the epidural space. Consequently, the reduction in intracranial pressure combined with the traction of pain-sensitive structures such as veins, meninges, and nerves leads to a persistent headache. This type of headache usually appears after 48-72 hours and is commonly distributed at the occipital areas, often radiating to the neck and shoulders. It is characterized by worsening in the standing or sitting position and mild relief while lying down. In addition, it may appear combined with other symptoms, such as nausea, vomiting, tinnitus, and vertigo [3,4].

Postdural puncture headache (PDPH) is commonly treated with bed rest, fluid administration, paracetamol, and non-steroidal anti-inflammatory drugs (NSAIDs), caffeine, synthetic adrenocorticotropic hormone ACTH, and corticosteroids. The epidural blood patch (EBP) represents another solution, which aims to administer autologous blood (10-20 mL) to the epidural space in the vicinity of the initial puncture to form a blood clot at the leak point. It should always be applied with extreme caution, as it could lead to other complications. The most frequent complications include failure (15-20%), PDPH worsening by accidentally creating additional dural tear(s), back pain, and infection. The latter requires urgent evaluation and care and is characterized by fever, malaise, erythema, and purulence at the injection site [3,4].

The purpose of this study is to report three cases where EBP was performed immediately after dural puncture and drug administration for lumbago sciatica. After searching PubMed using the keywords “epidural, blood patch, and dural puncture,” no results described immediate execution of a blood patch after dural puncture and drug administration.

Rhodes is the largest island in its administrative region. Patients who travel from small and remote islands to Rhodes for health issues cannot withstand further financial, physical, and emotional stress caused by the postponement of their treatment.

## Case presentation

The patients were informed in detail about the procedure’s technique and advantages and possible complications and their solutions, and they provided informed consent. In addition, they were informed of and approved of the reporting of their cases. The cases were three patients suffering from lumbago diagnosed clinically and confirmed by magnetic resonance imaging, who attended the Department of Anesthesiology and Pain Unit of the General Hospital of Rhodes, Greece for epidural analgesia between 2013 and 2017. The patient cases were as follows.

Case 1

A 72-year-old female patient with disk hernias at the L2-L3, L3-L4, and L4-L5 intervertebral space complained of pain in the lumbar area irradiating to the lower limbs that prevented her from walking. She was attending her third and final session in 18 months, with progressive improvement of her condition. The patient was on medication for hypertension (metoprolol 100 mg) and type II diabetes (metformin 850 mg). The patient regained her walking ability two weeks after the session, referring to moderate pain only when lifting heavy objects.

Case 2

A 41-year-old male patient with disk hernias at the L3-L4 and L4-L5 intervertebral space complained of pain and paresthesia of the left lower limb, which limited his everyday activities. The patient reported no comorbidities, and this was his first epidural session. The patient reported an absence of paresthesia ten days after the session and total pain regression three weeks after the session.

Case 3

A 55-year-old female patient with disk hernias at the L3-L4, L4-L5, and L5-S1 intervertebral space complained of pain and paresthesia of both lower limbs. The patient reported no comorbidities, and this was her first epidural session. The patient reported an absence of symptoms one month after the session.

All standard protocols were followed (meticulous sterilization of the puncture site and constant monitoring of arterial blood pressure, heart rate, oxygen saturation, and IV access). The blood test results (complete blood count, biochemical parameters, and prothrombin time) were normal for all patients.

The patients were placed in a seated position, and a standard 18G Tuohy needle epidural set was used, selecting the L3-L4 intervertebral space and the interlaminar route. The epidural space was determined using the loss of resistance to air technique with 3 mL of air. At the moment of the dural puncture, all the patients manifested severe headache and pain in the nuchal region (numeric pain intensity scale = 10), nausea, vomiting, and profuse sweating without hemodynamic disturbances. The needle was immediately extracted, the patients were placed in the lateral position, and a second epidural puncture was performed at a higher intervertebral space (L2-L3). Then, 10 mg of ropivacaine 2%, 0.05 mg of fentanyl, 16 mg of dexamethasone, and 5 mL of normal saline 0.9% were administered, followed by administration of 20 mL autologous blood taken in a sterile manner from the cephalic vein.

The patients were retained in the Post-Anesthesia Recovery Unit for one hour after the procedure, and 1 L of Ringer’s lactate was administered. All three patients presented a gradual improvement in their condition, while their headaches vanished entirely after 30 to 35 minutes. No hemodynamic or other changes were registered. Afterward, they were transferred to the ward for monitoring, instructed to stay in bed, and released the following day with instructions to avoid physical strain and exercise, rest, and drink liquids.

All three patients were under daily phone contact for 10 days and follow-up visits were scheduled two, three, and four weeks after the session. They did not manifest any complications and their lumbago symptoms regressed.

All the patients reported that, despite the initial fright and intensity of the PDPH at the moment of the dural puncture, they were satisfied with the overall outcome, as both the PDPH and their lumbago symptoms regressed and spared them further financial, social, and medical stress.

## Discussion

According to the literature, the effectiveness of steroids injected in the epidural space against lumbalgia sciatica is variable. A systematic review by Abdi et al. reported strong and moderate evidence regarding short- and long-term pain relief, respectively [5]. Other systematic reviews, as reported by Pandey [6], suggest otherwise, causing confusion among physicians. The patients reported here indeed presented mixed responses to the treatment: one patient registered notable relief within two weeks of the session but still experienced moderate pain with certain movements, in contrast to the other two, who reported complete pain regression. All the patients, however, reported that they were satisfied with the outcome. Age, the nature of lumbago, and the medication access route (interlaminar, transforaminal, or caudal) seem to have a certain role regarding the efficacy of the epidural steroid injection treatment, but other studies have produced inconclusive results [5,6].

The sudden onset of PDPH is often associated with pneumocephalus. However, it usually involves frontal headache localization combined with upper extremity numbness, visual disturbances, seizures, hemiparesis or hemiplegia, lethargy, cranial nerve palsies, and cardiovascular instability. Pneumocephalus treatment is symptomatic and usually dissolves in three to five days. The patients mentioned above, in contrast, manifested only severe headache and pain in the nuchal region, nausea, vomiting, and profuse sweating, without hemodynamic disturbances or nervous system defects. At the same time, PDPH was absent 30 minutes after the EBP. According to the literature, applying the loss of resistance technique with natural saline reportedly significantly decreases the incidence of dural puncture and minimizes the possibility of pneumocephalus insurgence in the case of dural puncture [7,8].

High spinal block or total spinal anesthesia involves the spread of local anesthetic affecting the spinal nerves above T4 and intracranially, respectively. The signs and symptoms of this condition include bradycardia, hypotension, upper limb numbness and weakness, shortness of breath, respiratory arrest, slurred speech, lethargy, and loss of consciousness. A tear of the dura mater has been associated with involuntary total spinal anesthesia after an epidural block due to the leak of the local to the subarachnoid space. For that reason, our patients were closely monitored and were not released from the hospital until the next day. Cases described in the literature report onset of the spinal block shortly after drug administration [9]. However, this was not the case in those patients where catheter placement with continuous drug administration was involved. These patients manifested spinal block five hours after the catheter placement, as the volume of the administrated medication was significantly lower. Most of the cases that presented spinal block reported symptomatology regression two to five hours afterward, with complete absence of symptoms after 24 hours. There have been cases where neurological complications persisted from one week to four years after the incident. Nevertheless, these involved patients with total spinal anesthesia with rapid onset, significant hemodynamic instability, and loss of consciousness. The patients in the present report did not manifest any of the symptoms mentioned above, and the possibility of cephalad spread of local anesthetic was excluded. Furthermore, the clot tapping the dural tear created by the blood patch would impede such progress [10-12].

PDPH treatment with an EBP has been a subject of debate due to the complications that may arise. A review by Boonmak and Boonmak concluded that EBP brings more benefit than more conservative treatment [13]. Concerning EBP complications, mild to moderate back pain has been commonly reported but usually resolves in days and is reportedly preferable to the PDPH. The risk of further puncture of the dura mater was minimal, as in all patients, the procedure was not technically difficult, and the first dural puncture was purely accidental. As mentioned, the injected blood may serve as an infection nidus. Despite representing a rare complication, it requires urgent care and evaluation. According to the literature, signs of infection after EBP are reported to appear three to five days later. For that reason, the patients were informed of the signs and symptoms involved to report them promptly in our cases. Furthermore, they were under close surveillance and instructed to contact the physicians if they experienced any of the aforementioned symptoms [3,13-15].

The optimal timing for applying an EBP remains unclear. The study by Scavone et al. investigated the efficacy of a prophylactic EBP administered through the epidural catheter in parturients after delivery, finding that an EBP seemed to shorten the duration of PDPH but not the incidence. Conversely, in the study by Stein et al., a prophylactic EBP decreased PDPH incidence. The difference can be attributed to the different PDPH treatment modalities of the second study. In a study by Armstrong et al., it was hypothesized that the success of an EBP is probably reduced if performed close to the time of the dural puncture.The authors speculated that this specific moment represents the peak of CSF leakage, and the relatively high volume of CSF would impede clot formation due to hemodilution. However, they also indicated that a larger blood volume could reduce the possibility of clot destabilization. Regarding the necessary volume of blood for successful EBP, the study by Paech et al. found that 20 mL of autologous blood seemed to be sufficient [16-20].

## Conclusions

Of course, a series of only three cases cannot result in a firm conclusion. However, dural puncture represents a complication that cannot be predicted, and although it is a frequent complication, it is relatively uncommon among experts, and its association with the rapid onset of PDPH is rare. The nature of the cases described above makes more extensive studies, such as randomized control trials, difficult and possible to complete only in the long term.

Nevertheless, as noted in the cases mentioned above, an EBP executed immediately after dural puncture seems to relieve PDPH fast and does not seem to impede epidural analgesia. Thus, the patient is immediately spared from PDPH symptoms and avoids the physical and financial stress of having to reschedule an analgesic session. Finally, an EBP saves time by not postponing analgesia that improves life quality. Further research could bring more precise conclusions.
